# COVID-19 in Southeast Asia: current status and perspectives

**DOI:** 10.1080/21655979.2022.2031417

**Published:** 2022-01-26

**Authors:** Dinh-Toi Chu, Suong-Mai Vu Ngoc, Hue Vu Thi, Yen-Vy Nguyen Thi, Thuy-Tien Ho, Van-Thuan Hoang, Vijai Singh, Jaffar A. Al-Tawfiq

**Affiliations:** a Center for Biomedicine and Community Health, International School, Vietnam National University, Hanoi, Vietnam; b Department of Natural Science and Technology, International School, Vietnam National University, Hanoi, Vietnam; c Thai Binh University of Medicine and Pharmacy, Thai Binh, Vietnam; d Department of Biosciences, School of Science, Indrashil University, Rajpur, India; e Infectious Disease Unit, Specialty Internal Medicine, Johns Hopkins Aramco Healthcare, Dhahran, Saudi Arabia; f Department of Medicine, Indiana University School of Medicine, Indianapolis, IN, USA; g Department of Medicine, Johns Hopkins University School of Medicine, Baltimore, MD, USA

**Keywords:** Southeast Asia, COVID-19, SARS-CoV-2, perspectives, omicron variant

## Abstract

Coronavirus Disease-2019 (COVID-19) has spread globally with catastrophic damages to the public health, social and economy since the beginning of the outbreak. In 2020, Southeast Asia proved that it could prevent the worst effects of a pandemic through the closure of activities and borders and movement restriction, as well as social distancing. Nevertheless, with the occurrence of the common variants of concern (VOCs), especially Alpha (B.1.1.7), Beta (B.1.351), Delta (B.1.617.2), Southeast Asia is facing a significant increase in the Severe Acute Respiratory Syndrome Coronavirus 2 (SARS-CoV-2) infections. Now, the area also has the threats of the spreading out of the dangerous variant – Omicron (B.1.1.529) from other close countries or regions. COVID-19 countermeasures such as closures and social distancing seem to be insufficient. Moreover, Southeast Asia is being held back by a shortage of vaccines and other medical resources. This work focuses on describing the COVID-19 situation, the virus variants, and the coverage of COVID-19 vaccination in the area. We also provide perspectives on the COVID-19 vaccine distribution, protecting the economic capitals, developing the green zone, and the importance of finding more vaccine supplies in Southeast Asia.

## Introduction

COVID-19 is an acute respiratory disease caused by a new SARS-CoV-2. It was first emerged on 31 December 2019 in Wuhan city, China [1]. On 13 January 2020 coronavirus infections outside China were reported

[2]. SARS-CoV-2 infections can range from asymptomatic to acute respiratory distress [3]. As of 17 September 2021 the World Health Organization (WHO) recorded 227,951,739 COVID-19 infections globally and 4,686,522 deaths.

The diversity of the SARS-CoV-2 was initially reported to be very low [2]. However, since the summer of 2020, new variants have been notified, and the WHO classifies new SARS-CoV-2 virus variants as variants of concern (VOCs) and variants of interest (VOIs). VOCs have the potential to cause increased transmissibility, increased virulence, altered clinical presentation, and reduced the effectiveness of social and public health measures as well as available diagnoses, vaccines, and treatments. The VOCs include Alpha, Beta, Gamma, and Delta, and now, Omicron [4]. A VOC could be increased transmission and mortality [5] and reduce the effectiveness of vaccines, therapies, or other medical measures. A VOI is a variant with a genetic capability that affects the characteristics of the virus [5].

Alpha (B.1.1.7) variant was first appeared in the United Kingdom (UK) [6]. It was reported to be 43% to 82% more transmissible, surpassing the previously existing variants to become the predominant SARS-CoV-2 variant in the UK [7]. Beta (B.1.351) was first found in South Africa. This variant increases transmission risk and reduces neutralization by monoclonal antibody therapy, convalescent sera, and post-vaccination sera [7]. Gamma (P.1) was first identified in travelers from Brazil to Japan, and Delta (B.1.617.2) was first found in India [8], spreading faster than other variants. The Delta has ten mutations that mutated protein [7], and this variant is up to 50% more transmissible than the Alpha variant [9]. Delta was also found in the United States just a few months ago but now accounted for more than 90% of all COVID-19 cases [10]. In addition, the Delta variants also became the most common variant in the UK [11]. On the other hand, VOIs including Eta, Iota, Kappa, Lambda [4] have genetic changes that affect viral characteristics such as transmissibility, disease severity, immune escape, diagnostic or therapeutic escape. In addition, these variants have been identified as a significant cause of community transmission or clusters of COVID-19 in multiple countries, with increasing case numbers over time and represent a new risk to global public health. Both VOCs and VOIs are very risky. Significantly, the most concerned variant of SARS-CoV-2 named Omicron (B.1.1.529), reported to WHO from South Africa on 24 November 2021 leads to a global urgent public health alert [12,13].

Suspected or confirmed cases of COVID-19 require precautions to be taken to minimize the spread of the disease to the surrounding community. Both the patient and people in contact must take precautions [14]. To avoid spreading the disease, confirmed or suspected COVID-19 patients should avoid direct contact with others. They were also recommended to wear a mask [14]. Aerosol therapy using a nebulizer while on mechanical ventilation can also be a source of the spread of the COVID-19 infection. The use of aerosol generators is therefore not recommended

[15]. In addition, methods to protect people from COVID-19 recommended by the Centers for Disease Control and Prevention [16] are: getting vaccinated, diligently washing your hands, stay away from crowds and places where the air is poorly ventilated. Moreover, covering coughs, sneezes by wearing a mask or using tissues, cleaning the house, household items, regularly monitoring the health are nonspecific preventive measures. In addition, the WHO also recommends if COVID-19 is spreading in the community, stay safe by taking simple precautions. Maintenance at a distance of at least 2 meters from others may reduce the risk of infection when they cough, sneeze, or speak. In case of suspected symptoms for COVID-19, contact medical centers are needed [17].

COVID-19 has caused a health crisis for the citizens in Southeast Asian countries, which are Brunei, Burma (Myanmar), Cambodia, Timor-Leste, Indonesia, Laos, Malaysia, the Philippines, Singapore, Thailand, and Vietnam. Their governments have policies to respond and manage risks as well as guidelines on vaccines for the citizens [18]. This review focuses on describing the COVID-19 situation in Southeast Asia. We also provide perspectives on the COVID-19 vaccine distribution, protecting the economic capitals, developing the green zone, and the importance of finding more vaccine supplies in these countries.

## COVID-19 epidemics in Southeast Asia

### Current status of COVID-19

At the end of December 2019 in Wuhan City, Hubei Province, China, ‘pneumonia of unknown cause’ cases continuously appeared [19]. Considering that information, the WHO requested the Chinese authorities provide information and conduct investigations to determine the cause of atypical pneumonia reported in Wuhan [20]. Through research, Chinese scientists have determined that the outbreak in Wuhan was caused by a new type of coronavirus [20]. They have found up to 79.5% gene sequence similarity with the previous SARS-CoV-1 [21]. This new coronavirus, named 2019-nCoV, is a new clade in the sarbecovirus subfamily, Ortho Corona Virinae. In the coronavirus family, SARS-CoV-2 is the seventh member [19]. On 11 February 2020 WHO announced an official name for the disease caused by the novel coronavirus as COVID-19. COVID-19 is the third outbreak of human coronavirus disease, following severe acute respiratory distress syndrome (SARS) and the Middle East Respiratory Syndrome Coronavirus (MERS-CoV) [22].

The first death of the coronavirus was indicated on 9 January 2020 [23]. Soon after, dozens of non-Chinese infections were reported, including France (23 January 2020) and Algeria (25 February 2020). The epidemic has spread to 123 countries and territories, with global infections exceeding 126,000 cases and more than 4 million deaths since the beginning of the outbreak [24]. On 11 March 2020 the WHO has issued a statement calling ‘COVID-19’ a ‘Global Pandemic’ [24].

Southeast Asia is also on the list of regions where the number of COVID-19 infections has significantly increased in recent times [25]. 13 January 2020 the first confirmed case of the virus in Southeast Asia is a 61-year-old woman from Thailand [26]. Shortly after that, other countries in Southeast Asia also confirmed the first cases of COVID-19, such as Vietnam on 23 January 2020.

[27], Malaysia on 25 January 2020 [28], Cambodia on 27 January 2020 [29], the Philippines on 30 January 2020 [30]. Nowadays, Southeast Asia has seen sharp increases in cases and deaths from the disease. As of 17 September 2021 Southeast Asia recorded 11,324,390 confirmed cases and 249,529 deaths [17]. The case fatality rate CFR in Southeast Asia countries was significantly higher compared to the rate worldwide (3.3% versus 2.1%, p < 0.0001, chi2 test, statistical analysis was carried out using OpenEpi and available data in WHO Coronavirus COVID-19 Dashboard (Table 1). Specifically, Indonesia recorded a total of 4,185,144 cases of SAR-CoV-2 infection, including 140,138 deaths (CFR = 3.3%) [31]. This country has the most significant number of people infected with COVID-19 in Southeast Asia, followed by the Philippines, with 2,040,343 cases and 33,873 deaths (CFR = 1.6%) [31]. Singapore and Laos are currently the two countries with the lowest CFR in Southeast Asia (0.1% and 0.1%, respectively) [31]. Interestingly, Myanmar recorded only 440,741 confirmed cases, but the CFR was highest in the region (3.8%).

**Table 1.** Status of COVID-19 pandemic in 11 countries of Southeast Asia

Country	Total cases	Total death	Case fatality rate	Rate of confirmed cases per 100,000 population	Percentage of people fully vaccinated against COVID-19	Percentage of people with at least 1 dose	Available vaccines
Myanmar	440,741	16,869	3.8	804.2	5.9	9.0	Oxford/AstraZeneca, Sinopharm/Beijing
Indonesia	4,185,144	140,138	3.3	1514.4	15.8	27.7	Moderna, Oxford/AstraZeneca, Pfizer/BioNTech, Sinopharm/Beijing, Sinovac
Vietnam	656,129	16,425	2.5	668.4	6.1	26.8	Moderna, Oxford/AstraZeneca, Pfizer/BioNTech, Sinopharm/Beijing, Sputnik V
Cambodia	102,834	2089	2.0	606.8	59.0	68.0	Johnson&Johnson, Oxford/AstraZeneca, Sinopharm/Beijing, Sinovac
Philippines	2,324,475	36,328	1.6	2093.2	11.3	16.8	Johnson&Johnson, Moderna, Oxford/AstraZeneca, Pfizer/BioNTech, Sinovac, Sputnik V
Malaysia	2,049,750	22,355	1.1	6253.8	54.8	66.5	CanSino, Oxford/AstraZeneca, Pfizer/BioNTech, Sinovac
Thailand	1,448,792	15,124	1.0	2071.2	19.0	39.7	Oxford/AstraZeneca, Pfizer/BioNTech, Sinopharm/Beijing, Sinovac
Timore-Leste	18,943	103	0.5	1409.6	17.4	30.4	Oxford/AstraZeneca, Sinovac
Brunei	4675	23	0.5	1058.8	33.7	53.7	Oxford/AstraZeneca, Sinopharm/Beijing
Laos	18,059	16	0.1	244.7	25.2	35.9	Johnson&Johnson, Oxford/AstraZeneca, Pfizer/BioNTech, Sinopharm/Beijing, Sinovac, Sputnik V
Singapore	74,848	59	0.1	1269.3	77.0	78.9	Moderna, Pfizer/BioNTech, Sinovac
Southeast Asian	**11,324,390**	**249,529**	**3.3**	**1677.4**	**29.6**	**41.2**	-
World	**227,951,739**	**4,686,522**	**2.1**	**2894.6**	**31.1**	**42.8**	-

Note: *Data were extracted from Worldometers (https://www.worldometers.info/coronavirus/), Our World in Data (https://ourworldindata.org/covid-vaccinations?country=OWID_WRL) as of 17 September 2021 and World Population Review (https://worldpopulationreview.com/continents/asia-population*).

### The variants of SARS-CoV2

In the current SARS-CoV-2 pandemic, countries in Southeast Asia have the common occurrence of three strains: Alpha (B.1.1.7), Beta (B.1.351), and Delta (B.1.617.2), which were circulating in original countries, then and rapidly infected people in neighboring countries and worldwide [32]. At the end of May 2020, one study analyzed 444 SARS-CoV-2 genome sequences available on the GISAID platform from 6 Southeast Asian countries. The author showed that most of the mutations found in this region were also prevalent in North America and European countries, suggesting a possible transmission route [33]. Like the other parts of the world, this area is facing the threat of the most recent and concerning variant – Omicron, which was first discovered in South Africa on 24 November 2021 [12]. Still, now on December 03, it is spreading to more than 24 countries in the world, including the countries or regions close to Southeast Asia such as India, Japan, South Korea, and Hong Kong.

Between March 2021 and June 2021, Cambodia and Thailand recorded two VOCs, Alpha and Delta [34]. In addition, the Beta variant was found in Indonesia and Malaysia. Meanwhile, in Laos, the least affected country by COVID-19, worrisome cases of infected people have begun to appear [32]. In Malaysia’s 3^rd^ wave (September 2020) of infections, a new variant named B.1.524 appeared [21]. Two spike protein mutations, including a common D614G and A701V, have been discovered [35]. A recent report described nine variants of SARS-CoV-2 with the D614G mutation in Malaysia [36]. One study reported that variant B.1.466.2 came from Malaysia and B.1.468 from Indonesia [36]. Another study identified the SARS-CoV-2 strain circulating mainly in Malaysia as strain B, common in East Asia. The other two lines are strain C originating in European countries and strain A from the US and Australia [37]. A study analyzing SARS-CoV-2 genomes indicated that SARS-CoV-2 variants originated from Europe and Asia in Indonesia. The study also determined the high prevalence of the D614G mutation in the country [38].

Delta (B.1.1.7) is considered an important variant with the ability to infect far beyond the previous Alpha strain [32]. It has been present in 135 countries, including Southeast Asian regions such as Vietnam, Indonesia, Malaysia, and Thailand [32,39]. In March 2020, Vietnam recorded COVID-19 cases of the Delta variant [40]. In January 2021, infections of the B.1.1.7 variant were recorded in 3 provinces of Indonesia [41]. Delta variant was spread quickly after 2 months from India to Indonesia, which is a significant cause of the record-high number of COVID-19 infections in other countries of this region [42]. The newest variant, the Omicron (B.1.1.529) is reported to be much faster in terms of spreading than the Delta and other variants, with the doubling time shortened to 2 to 3 days [43]. The rate of Omicron infection was found to be 3.2 times higher than the Delta [44]. This variant has been discovered in 9 out of 11 countries in Southeast Asia, with total cases of 2,016 (data taken from 31 December 2021) [45].

**Figure 1.**
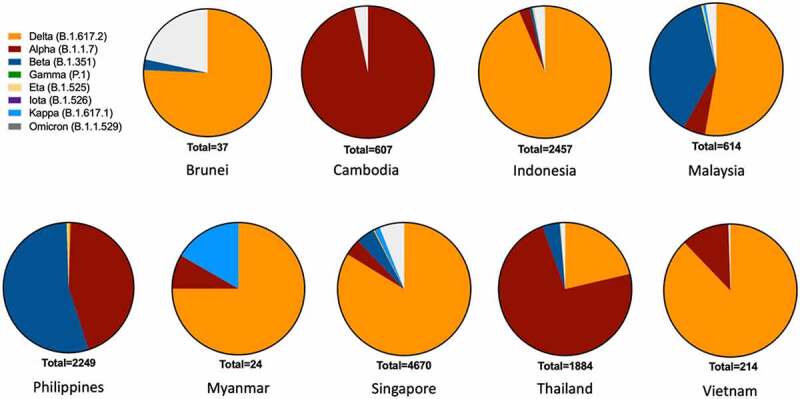
**The presence of COVID-19 variants in Southeast Asia**. The graph describes the total number of COVID-19 variants in each Southeast Asian country, in which, Brunei (37), Cambodia (607), Indonesia (2457), Malaysia (614), Myanmar (24), Philippines (2249), Singapore (4670), Thailand (1884) and Vietnam (214). The data of Laos and Timor-Leste was not recorded. Data updated to 4 September 2021 Omicron upated on 31 December 2021. Variants are classified according to WHO (https://www.who.int/en/activities/tracking-SARS-CoV-2-variants/). The total number of variants in each country is based on GISAID Southeast Asia data (https://www.gisaid.org/hcov19-variants/).

Currently, all three variants, the Alpha, Beta, and Delta, are available in Myanmar. Alpha originated from Singapore and Thailand, while the other two strains came from the East of Thailand and the West of Bangladesh and India [32]. Variant B.6 was found in samples from Singapore and Malaysia. Two variants, B.1.36.1 and B.1.1, came from India, and one variant, B.1.80, was found in China. Thus, the second transmission phase (August-September 2020) in Myanmar may have originated from the B.1.36 variant since it was found in all tested samples [46]. A study showed that the overall CFR of COVID-19 in Singapore is lower than that in Japan. It suggests that the circulating SARS-CoV-2 variant in Singapore was associated with a decrease in severity [47]. In March 2020, a new variant, D614G in Spike protein, was identified. During the first 6 months, Singapore and South Korea were the only two countries in the world that did not report this type of mutation [48]. A study in Singapore compared the pathogenicity of three VOCs, alpha, beta, and delta, which beta variant is the most dangerous that deserves attention in this country for its lack of oxygen, high ICU treatment rate, and mortality [49] (Figure 1).

### The COVID-19 vaccination

**Figure 2.**
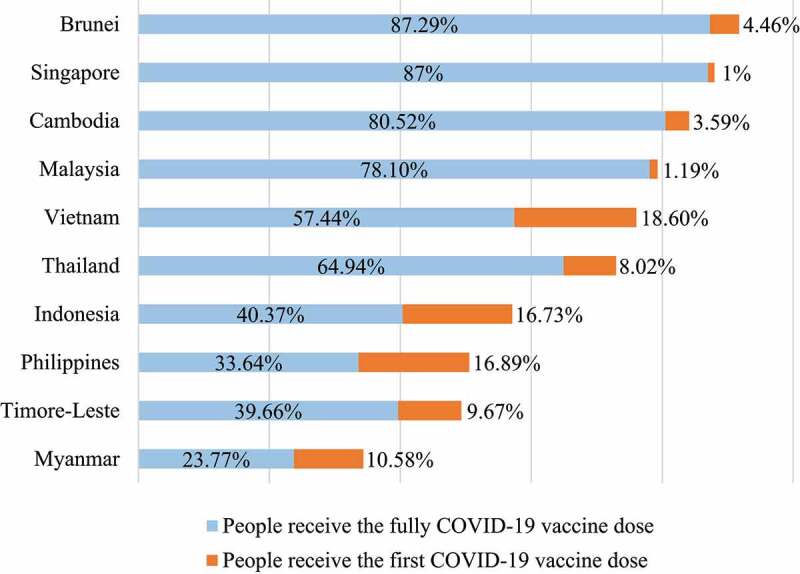
**The COVID-19 vaccinations in Southeast Asia**. The data updated to 28 December 2021 (https://ourworldindata.org/covid-vaccinations). The COVID-19 vaccination data of Laos was not recorded.

In the face of a complicated pandemic, vaccines play an important role in ending the COVID-19 pandemic. At the time of writing, 42.8% of the world’s population has received at least one dose of the COVID-19 vaccine [50]. However, the distribution of vaccines is uneven across countries (Table 1), depending on many factors such as the income levels, population size, vaccines allowed by the Government, as well as the ability to produce vaccines locally (Figure 2). For example, only 1.8% of people in low-income countries have received at least one dose of the vaccine [51]. Specifically, as of 17 September 2021 77% of the population were fully vaccinated against COVID-19 in Singapore [51]. Two other countries in Southeast Asia that also have a high percentage of their population fully vaccinated against COVID-19 are Cambodia with 59.0% and Malaysia with 54.8%. The coverage of the COVID-19 vaccine was lowest in Vietnam and Myanmar, with 6.1% and 5.9% population fully vaccinated, respectively (Table 1) [51]. Interestingly, Table 1 shows that CFR is high in countries with low vaccine coverage, such as Myanmar, Indonesia, and Vietnam (Table 1).

According to the most recent data, on 17 September 2021 29.6% of the Southeast Asian population were received the full dose of the vaccine. This rate is lower than the world with 30.4% [50]. Singapore is the leading country, with 77.0% of the population being vaccinated with full doses of vaccine, deploying 4.65 million doses, and 4.5 million people are fully vaccinated [51,52]. 59.0% and 54.8% of the total population in Cambodia and Malaysia received the full dose, respectively [51,52]. Cambodia has provided 19.2 million vaccine doses, in which 11.6 million people out of the country’s total population have been fully vaccinated [51,52]. Malaysia has vaccinated more than 21 million doses, of which 18.1 million people have met the number of injections [51,52]. Laos and Brunei have 25.2% and 33.7% of the total fully vaccinated population, respectively. Laos has injected 2.7 million doses, of which 1.9 million people are fully vaccinated [51,52]. In addition, Brunei has the advantage of a smaller population, provided a total of 237.015 doses of the vaccine, of which 148.587 people were fully vaccinated [51,52]. Other countries in Southeast Asia with a percentage of the total population fully vaccinated are Indonesia (15.8%), the Philippines (11.3%), and Thailand (19.0%) [51,52]. Indonesia’s population growth rate changes rapidly and has provided 77.4 million vaccine doses, but only 44.1 million people have been fully vaccinated [51,52]. The Philippines and Thailand have significantly lower populations, with 18.7 million and 28.4 million vaccine doses, meeting vaccine requirements for 12.6 million and 14.3 million people, respectively [51,52]. Vietnam and Myanmar are at the bottom of the list, with only 6.1% and 5.9% of the total population fully vaccinated [51,52]. Vietnam has now provided 26.3 million doses of vaccine, with 6.0 million people vaccinated with 2 doses [51,52]. In Myanmar, 4.9 million doses of vaccine are being deployed to the people, of which 3.2 million people are guaranteed to be fully vaccinated [51,52]. Most Southeast Asian countries have access to vaccines, including Pfizer/BioNTech, Moderna, Oxford/AstraZeneca, Jassen (Johnson & Johnson), Covishield, Sputnik V, Sinopharm (Beijing), and Sinovac [51,52]. The Philippines uses some other products such as Sinopharm (Beijing, Wuhan), Sputnik (V, Light), and Covaxin [51,52] (Table 1). With a global shortage of vaccines, low – and middle-income countries in Asia have yet to deploy full doses to the entire population. However, this is considered the most effective method at the moment [53]. A significant reason for vaccine shortages in Southeast Asian countries is affordability and supply chain constraints. All countries need a vast amount of vaccine, making it challenging to access manufacturers in low and middle-income countries. There is an inequality in access to vaccine resources in different parts of Southeast Asia. Most countries in Southeast Asia are interested in finding cheap vaccine manufacturers, investing in many cold storages with a maximum temperature of −70 to −80°C to store vaccines, allowing equal access to vaccines by people in different regions of the country

[54].

## Perspectives for controlling COVID-19 in Southeast Asia

### Effective delivery and the use of COVID-19 vaccine

In 2020, Southeast Asia proved that it could prevent the worst effects of the pandemic through the closure and movement restriction, as well as social distancing [55,56]. Nevertheless, with the increase in highly infectious variants nowadays, these measures seem insufficient to prevent COVID-19 effectively. Southeast Asia needs robust vaccination programs to keep up with new variants. However, Southeast Asia is being held back by a shortage of vaccines and other medical resources. Low-income countries in Southeast Asia will need significant support from external donors, not only for vaccines but also for accelerating vaccination. The International Federation of Pharmaceutical Manufacturers & Associations [57] had estimated that more than three billion doses of the vaccine would be produced worldwide by the end of June 2021 [57]; however, developed countries have monopolized the vaccine. As of mid-May 2021, only 0.3% of all vaccines used globally went to low-income countries [58]. Southeast Asia has been altogether subject to outer providers for its immunization programs.

China immediately arose as one of the most supportive source nations for the area, with the Sinovac vaccines in Indonesia in December 2020. Other worldwide vaccine makers such as the USA, European Union, Japan, and Australia are also mentioned with direct orders from Southeast Asia and the COVAX initiative [59]. The COVAX facility was established to promote the development and production of the COVID-19 vaccine and ensure fair and equitable access to all countries in the world [60]. Low-income countries are supported by COVAX Advance Market Commitment (COVAX/AMC) [61]. Cambodia, Indonesia, Laos, Myanmar, Philippines, Timor-Leste, and Vietnam are qualified nations under the COVAX/AMC arrangement [57,62]. Furthermore, Southeast Asia is additionally moving to decrease its reliance on outer makers. Vietnam, Indonesia, and Thailand are developing their vaccines [9,63,64]. Utilizing all mechanisms to get the vaccine is essential for the time being in Southeast Asia. Moreover, countries can consider buying highly effective vaccines made in Southeast Asia, such as vaccines from Vietnam.

On the other hand, effective vaccines and rational distribution are also leading concerns in Southeast Asia. States should consider the vaccine to priority target groups to ensure that the country is stable during a long-term epidemic. The priority group should be the army, healthcare providers, and other essential workers, with whom the persons are more likely to spread this disease due to living profiles and their work. In the next phase, the vaccine should be given to the elderly over 65 years old, people aged 18–64 years with comorbidities or with high-risk for severe infection, and non–healthcare essential workers. However, one obstacle is that non-healthcare essential workers are usually people with low economic conditions, such as garbage collectors, shippers, and supermarket checkout workers. To sum up, like the rest of the world, Southeast Asia will have to make complex changes to live with COVID-19. They also need a clear plan and strategy to avoid wasting vaccine resources. Furthermore, the economic, ethical, and epidemiological considerations are essential. It is necessary to have multi-sectoral coordination and multi-national coordination to use vaccines economically and effectively.

### Protection of the most important economic and administrative capitals

**Figure 3.**
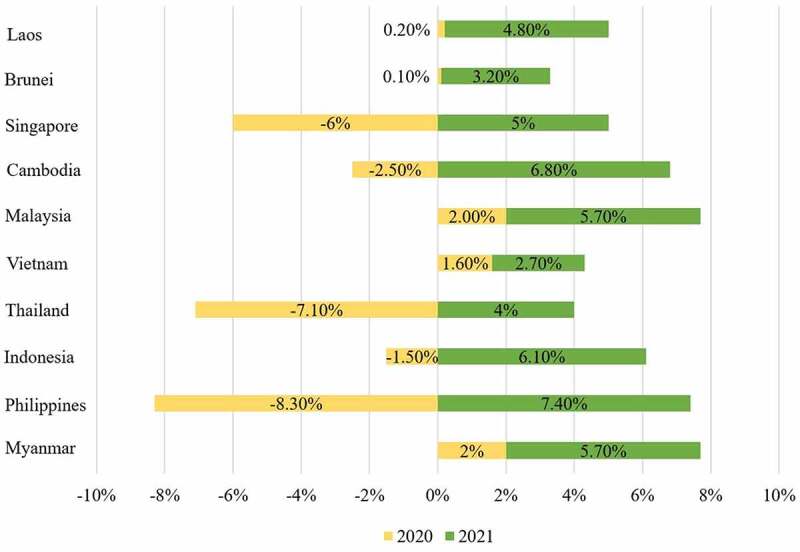
**Forecasted GDP growth rate in Southeast Asia by country from 2020 to 2021**. Data source (https://www.statista.com/statistics/621011/forecasted-gross-domestic-product-growth-rate-in-southeast-asia-2017/).

Shaken by the pandemic and its economic costs, Southeast Asia faced a decade of uncertainty after a long period of stability and relative economic growth (Figure 3). Waves of COVID-19 undermined Thailand’s economy in the first half of 2021. This pandemic has caused a 2.6% economic contraction in the first quarter of 2021 after a 6.1% decline in GDP in 2020 in Thailand. This is one of the steepest declines among the Association of Southeast Asian Nations (ASEAN). Meanwhile, the exports of services fell 74.8% yearly as Thailand’s border remained closed to most tourists [17,64]. Cambodia alone lost an estimated $3 billion in tourism revenue in 2020. According to the Minister of Tourism in Cambodia, Cambodia’s most visited tourist destination saw a 45.6% decline in tourist numbers in April 2020 and a 99.6% decline year on year [65]. About 2,956 businesses are associated with tourism, leaving 45,405 unemployed [65]. A total of 433 tourism-related companies and enterprises across the country have been temporarily closed. Vietnam’s Gross Domestic Product (GDP) increased by only 1.81% in the first haft of 2020, the lowest level in the past 30 years [66].

The road to recovery in most countries in this region will be long and difficult. The speed of economic recovery in Southeast Asia will depend on the global trajectory of the pandemic and the success of vaccine development. Therefore, it is necessary to focus on protecting the most important economic and administrative capitals, the economic locomotives of countries, which play a crucial role in their economy. If each country’s financial and administrative capital falls to the COVID-19 outbreak, the whole country will fall. Southeast Asian countries should look for vaccines as much as possible in the administrative capitals and the most important economic. The support of governments worldwide for health and vaccination programs in Southeast Asia seems to be the best investment to save lives and ensure continued economic recovery and societal stability in the region.

### Development and protection of the green zones

Green zoning COVID-19 areas have been considered an efficient way to limit the spread of coronavirus infection. This method uses colors to illustrate the epidemiological conditions of a territory. For example, green zones indicate that the epidemic is under control, while red zones point out the opposite. At first, the green zoning method was being applied in hospital wards, where they could mark the patients with COVID-19 infections (red zone) and areas for healthcare providers, as well as medical equipment (green zone) [67]. Now, we can see the applications of green zones as a COVID-19 indicator in a country or region [68]. Green zoning consists of four key steps, which are (1) divide each country/city into smaller zones, (2) use common epidemiological to evaluate the zone is either green or red, (3) have suitable public health measures depending on the zone’s color, and (4) permit transporting within green zones, but with a particular health condition (fully vaccinated, negative on the COVID-19 test, etc.) [69].

**Figure 4.**
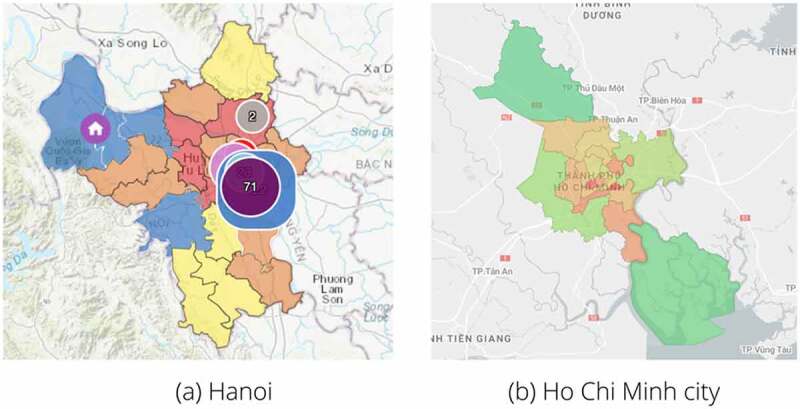
**A demonstration of the marking of the green zone areas in Hanoi** (a) **and Ho Chi Minh city** (b). (a) In Hanoi, the green zones are designated as ‘blue’ (0 cases of COVID-19). Yellow indicates 1–5 cases of COVID-19; Orange indicates 6–20 cases, and red shows more than 20 cases. The figure was taken from (https://covidmaps.hanoi.gov.vn/?page=home), data was shown as of 3 September 2021. (b) Ho Chi Minh city uses green, Orange, and red color indicators. There is also an appearance of the ‘nearly green zone,’ which are areas with very few cases of COVID-19. The figure was taken from (https://bando.tphcm.gov.vn/), data was recorded as of 3 September 2021.

For example, in Vietnam, Hanoi and Ho Chi Minh City are the only cities that apply green and red zones because these are the two major cities in Vietnam. The zones are being divided according to the districts (Figure 4). Within the green zones, public places are allowed to be opened, and people are freely traveling inside the green areas, with mandatory use of masks. Individuals transporting from the red zone must have government-issued paper, as well as a negative test of SARS-CoV-2 within the last 72 hours or have already got 2 doses of COVID-19 vaccines.

Green zones contribute a significant impact for eradicating COVID-19 in Southeast Asia. Therefore, it is crucial to develop and protect the green zones, both regional and national. Especially with the rapid spreading of the Omicron variant, green-zoning is one of the critical factors to prevent the increase of this variant since its likelihood of direct infection is 21.6%. In comparison, the Delta is 10.7% [44]. Development and protection of the green zones could mean: (1) Restrict traveling from red zone areas to green zone areas and strictly monitor travelers from the red zones. In order to permit inside the green zones, people are coming from contaminated sites are required to take the COVID-19 test at least 48 hours before traveling, with negative results [70] or need to be fully vaccinated against the coronavirus. Furthermore, quarantine may be of value in limiting the spread of the virus to green zones regions, which are 14–21 days for people who tested negative for coronavirus and 7 days for fully vaccinated people. During quarantine, patients needed to be taken care of and observed by medical experts and tested with COVID-19 several times. And (2) Restrict activities within the green zone regions. Although the green zones are marked as the ‘save zones’ with manageable cases of COVID-19, it is critical to note that there will be possible occasional outbreaks. However, public places are allowed to be opened with a limited of people. Furthermore, all citizens in the green zones should be required to wear masks when they are going out and comply with all the safety measures for protecting themselves from COVID-19, such as washing hands, avoiding touching face, and social distancing.

### Seeking more vaccine supplies and developing domestic vaccines

With the high demand for vaccination domestically, scientists in Southeast Asia are sparing no effort in developing domestic vaccines. However, until the present day, only four Southeast Asia are researching and developing their vaccine: Vietnam, Thailand, Indonesia, and Singapore. The Minister of Research and Technology in Indonesia is developing a domestic vaccine, an S subunit vaccine named ‘Merah Putih’ [56]. However, this vaccine is still pre-clinical, estimating phase 3 clinical trials in 2022 [63]. Thailand is about to launch its vaccine using mRNA technology, the ‘ChulaCov19’. It has been shown to have a promising result on mice and primate models, and phase 1 of the clinical trials was planned for September 2021 [71]. The Mahidol University in Thailand is also developing a vaccine called ‘Butanvac,’ in phase 2 of the clinical trials in Thailand and Brazil [72]. The COVID-19 vaccine developed from Singapore, ‘Lunar-COV19,’ uses mRNA technology. This vaccine can elevate titers of neutralizing antibodies after a single injection [73]. In Vietnam, Nanogen Pharmaceutical Biotechnology JSC is developing the ‘Nanocovax’ vaccine. The vaccine has entered phase 3 of the clinical trials in June 2021 and is expected to be available in the fourth quarter of 2021 [74,75]. Another vaccine is under clinical trial in Vietnam, the ‘Covivac’ vaccine [71]. Table 2 summarizes all domestic vaccines developed in Southeast Asia (Table 2).

**Table 2.** The recent development of the COVID-19 vaccine candidates in some countries in Southeast Asia

Country	Developer	Vaccine	Vaccine technology	Clinical trial phase
Indonesia	Biofarma, Ejikman Institute and Indonesian Insitute of Science	Merah Putih	Subunit S	Pre-clinical trials, phase 3 estimated in 2022
Thailand	Chula Vaccine Research Center at Chulalongkorn University	ChulaCov19	Self-replicating mRNA	Phase 1 completed, planning to conduct phase 2
Thailand	Mahidol University	Butanvac	Non-replicating viral vector	Phase 2 in Thailand and Brazil
Singapore	Duke-National University of Singapore, Arcturus Therapeutics	Lunar-COV19	Self-replicating mRNA	Phase 1 and 2
Vietnam	Nanogen Pharmaceutical Biotechnology JSC	Nanocovax	Subunit vaccine	Entered phase 3 of clinical trials, expected to be available on the fourth quarter of 2021
Vietnam	Institute of Vaccines and Biological Medical – Nha Trang	Covivac	S protein	Entered phase 2 clinical trial

As of 17 September 2021 Southeast Asia has recorded a total of 11,324,390 cases of COVID-19 [31], and this number is increasing every day. Therefore, there is an immediate demand for vaccines among Southeast Asian countries. Because all the domestic-developed vaccines in Southeast Asia are still undergoing clinical trials, finding the vaccine sources from outside is an instant needed. Southeast Asia majorly depends on the COVAX program for its vaccine demands. The potential disadvantage of COVAX is that high-income countries will be the first in line for COVID-19 vaccines once distributed due to the Advance Market Commitment (AMC) agreement. Low – and middle-income countries access to the COVID-19 vaccines will be undermined [76]. As a result, it is essential for Southeast Asian countries to self-develop and distribute their vaccines. Moreover, requesting for transferring the vaccine technology from developed countries is also crucial for boosting the development and manufacture of COVID-19 vaccines in Southeast Asia. Countries that want to develop cannot completely close their borders. With the opening of trade, virus transmission is inevitable. Therefore, preventing the pandemic is a shared global task.

### Conclusion and future outlook

Since the end of 2019, the world has suffered from a global pandemic, the COVID-19. Until today, 4 VOCs and 5 VOIs of the SARS-CoV-2 have been found, each contaminating and harming the human host in different ways. In 11 countries of Southeast Asia, over 11 million people are infected with this virus, and this number is increasing every day. Therefore, administering vaccines for citizens is urgent. The primary source of vaccines for Southeast Asian countries is through the COVAX program. However, the existing challenge is that high-income countries will be first in the vaccine lines, whereas most Southeast Asian countries are middle – and low-income. With a limited source of vaccines, it is necessary to divide people into groups of prioritizing, determining who will take the shot first. Furthermore, the fight to combat this pandemic will be long and arduous. It is essential to protect the economic and administrative capitals. Moreover, to limit the spread of COVID-19 in the country, division of the regions into red and green zones to control the epidemic is also needed. Finally, Southeast Asia is at the bottom of the COVAX program, and with the high vaccine demand, it is crucial to develop domestic-made vaccines. Nevertheless, since most of the self-developed vaccines in Southeast Asia are still only in phases 1 and 2 of the clinical trials, there is immediate importance of finding outside vaccine supplies to push the vaccination among the countries faster and more efficiently.

## Research highlights

- COVID-19 has spread globally with catastrophic damages to the public health, social, and economics

- Southeast Asia is facing a significant increase in SARS-CoV-2 infections as the occurrence of several virus variants such as Alpha (B.1.1.7), Beta (B.1.351), and Delta (B.1.617.2).

- Southeast Asia also has dangerous threats by the spreading out of the Omicron (B.1.1.529) variant.

- More COVID-19 vaccine coverage, protecting the economic capitals, and developing the green zone are needed for Southeast Asia.

## Abbreviation

AMC: Advance Market Commitment
ASEAN: Association of Southeast Asian Nations
CDC: Center of Disease Control and Prevention
COVAX: COVID-19 Vaccines Global Access
COVAX/AMC: COVAX Advance Market Commitment
COVID-19: Coronavirus Disease-2019
GDP: Gross Domestic Product
GISAID: Global initiative on sharing avian influenza data
IFPMA: International Federation of Pharmaceutical Manufacturers & Associations
SARS-CoV-2: Severe acute respiratory syndrome coronavirus 2
UK: United Kingdom
USA: United States of America
VOCs: Variants of concern
VOIs: Variants of interest
WHO: World Health Organization
